# Comparative evaluation of the physicochemical properties of nano-hydroxyapatite/collagen and natural bone ceramic/collagen scaffolds and their osteogenesis-promoting effect on MC3T3-E1 cells

**DOI:** 10.1093/rb/rbz026

**Published:** 2019-10-05

**Authors:** Xiongxin Lei, Jianping Gao, Fangyu Xing, Yang Zhang, Ye Ma, Guifeng Zhang

**Affiliations:** 1 National Key Laboratory of Biochemical Engineering, Institute of Process Engineering, Chinese Academy of Sciences, Beijing 100190, China; 2 School of Chemical and Engineering, University of Chinese Academy of Sciences, Beijing 100049, China; 3 College of Food Science and Engineering, Shandong Agricultural University, Taian 271018, China; 4 School of Life Science and Technology, Henan Institute of Science and Technology, Henan, 453003, China; 5 Department of Pathogen Biology and Immunology, School of Basic Course, Guandong Pharmaceutical University, Guangzhou, 510006, China

**Keywords:** nano-hydroxyapatite, natural bone ceramic, collagen, scaffold, MC3T3-E1, osteoblasts

## Abstract

The use of various types of calcium phosphate has been reported in the preparation of repairing materials for bone defects. However, the physicochemical and biological properties among them might be vastly different. In this study, we prepared two types of calcium phosphates, nano-hydroxyapatite (nHA) and natural bone ceramic (NBC), into 3D scaffolds by mixing with type I collagen (CoL), resulting in the nHA/CoL and NBC/CoL scaffolds. We then evaluated and compared the physicochemical and biological properties of these two calcium phosphates and their composite scaffold with CoL. Scanning electron microscopy (SEM), X-ray photoelectron spectroscopy (XPS), Fourier-transform infrared spectroscopy (FTIR), X-ray diffraction spectroscopy (XRD) and compressive tests were used to, respectively, characterize the morphology, composition, distribution and the effect of nHA and NBC to collagen. Next, we examined the biological properties of the scaffolds using cytotoxicity testing, flow cytometry, immunofluorescence staining, biocompatibility testing, CCK-8 assays and RT-PCR. The results reflected that the Ca^2+^ released from nHA and NBC could bind chemically with collagen and affect its physicochemical properties, including the infrared absorption spectrum and compression modulus, among others. Furthermore, the two kinds of scaffolds could promote the expression of osteo-relative genes, but showed different gene induction properties. In short, NBC/CoL could promote the expression of early osteogenic genes, while nHA/CoL could upregulate late osteogenic genes. Conclusively, these two composite scaffolds could provide MC3T3-E1 cells with a biomimetic surface for adhesion, proliferation and the formation of mineralized extracellular matrices. Moreover, nHA/CoL and NBC/CoL had different effects on the period and extent of MC3T3-E1 cell mineralization.

## Introduction 

Various systemic bone defects can arise from wounds, tumors, aging or other factors, and are a common clinical disease. The treatment for this condition is repair of the tissue, which commonly involves autogenous bone grafting and allogeneic bone grafting. Autografts are regarded as the gold standard for the repair of large bone defects, however this causes a secondary injury in order to obtain donor bone. Limited supply is also a problem. Another option for repair is allograft, but this poses a real risk of disease transmission from donor to recipient [1, 2]. These aforementioned shortcomings for autografts and allografts, along with the large demand for bone grafts, have resulted in the emergence of bone tissue engineering technology, which is expected to overcome these problems and put forth a promising treatment strategy. Currently, a variety of graft substitutes or matrix materials have evoked the enthusiasm of researchers. These include natural and synthetic polymers, ceramics and composites [3]. Bone is a natural organic-inorganic ceramic composite that consists of collagen fibrils and contains embedded, well-arrayed, nano-crystalline, rod-like inorganic materials [4, 5]. Thus, organic–inorganic composite scaffold materials have been the focus of recent research efforts. Hydroxyapatite (HA) has the general formula Ca_10_(PO_4_)_6_(OH)_2_ and is chemically similar to the inorganic component of bone matrix. This characteristic has led to its wide usage in bone tissue engineering materials [6, 7], as its similarity to natural bone matrix makes it particularly appealing [8, 9]. The main advantages of synthetic HA are its biocompatibility, slow biodegradability *in situ* and good osteoconductive and osteoinductive capabilities [10, 11]. By far, synthetic HA has been used most widely for the repair of hard tissues, such as bone repair, bone augmentation, as well as for the coating of implants or to act as filler in bone or teeth [12]. Nano-hydroxyapatite (nHA) has also been a good candidate as a bone substitute for biomedical applications. Among other beneficial mechanical properties, its greater surface area can allow for enhanced densification, which may also improve fracture toughness [11]. Moreover, when compared to coarser crystals, nHA is expected to have better bioactivity [13]. Importantly, one study has demonstrated that nanostructure composites can enhance *in vitro* and *in vivo* osteoblast functions, including adhesion, proliferation, synthesis of bone-related proteins and the deposition of calcium-containing mineral [14].

Recently, another widely studied alternative bone substitute has been highlighted: xenogenous bone. Morphologically and structurally similar to human natural bone, xenogenic bone is usually of bovine origin and it is easy to obtain, has a lower cost and is available in unlimited supply. It consists mainly of collagen and HA [15, 16]. High temperature sintering has been suggested as an alternative process for obtaining protein-free bovine bone, which is usually called natural bone ceramic (NBC) [15, 16]. The crystalline phase composition of NBC is similar to that of natural bone mineral, which has an HA composition of about 93 wt% and a β-tricalcium phosphate (Ca_3_(PO_4_)_2_, β-TCP) composition of about 7 wt% [17]. In addition, heat-treated xenogenous bone has an inter-connective porous structure (up to about 70 vol% porosity) that is similar to that of natural bone trabeculae, which allows for faster bone in-growth within the defected parts of the bone [18]. Moreover, the HA powder derived from bone also has excellent biocompatible and osteoconductive properties, which lends it great potential as a bone substitute [19–21].

Despite these advantages, certain factors restrict the clinical applications of HA ceramics, such as low mechanical strength, lack of adhesion between particles, poor clinical maneuverability and defects such as easy dispersion after implantation [22]. Therefore, a ceramic powder that can, at the point of application, be molded into a specific form by ‘adhesion’ is necessary to advance the therapeutic options for bone repair.

Type I collagen is the most abundant and best-studied collagen, especially bovine tendon type I collagen (CoL) which is extensively applied in clinical applications due to its good biocompatibility and absorbability [23–25]. More than 90% of the organic mass of bone and collagen fibrils contributing to the structural backbone of bone are comprised of CoL [26, 27]. Its special triple helix structure has the effect to induce mineral deposits, moreover, its surface contains osteoblast adsorption sites and mineral deposits, which, when used as a scaffold, can effectively enhance the mineralization process and generate new bone in the implantation [28]. Considering its biological properties and large molecular weight (about 30 kDa), collagen gel can be used as an adhesive for powder material. Recently, CoL has been widely used as a scaffold in skin and bone tissue engineering [25, 29].Typically, when collagen is used as tissue engineering scaffold, cross-linking is executed to enhance its mechanical strength and resist enzymolysis *in vivo*.

In this study, two kinds of HA particles, nHA and NBC, were mixed with CoL hydrogel to prepare a bio-composite hydrogel. Following a series of processing steps, such as molding, freeze-drying and glutaraldehyde cross-linking, we obtained two composite scaffolds, nHA/CoL and NBC/CoL. We then assessed their surface characteristics, morphologies and mechanical properties. We further studied them with *in vitro* experiments to evaluate their biological properties, especially the osteo-promoting effect of the scaffold to MC3T3-E1.

## Materials and methods

### Materials

Type I collagen was purchased from Collagen Biotechnology Co. Ltd. (Hebei, China); synthesized nHA (≥97%, <100 nm particle size) was purchased from Aladdin (USA); MC3T3-E1 cells were provided by the National Infrastructure of Cell Line Resource (China); alpha minimum Eagle’s medium (α-MEM), fetal bovine serum (FBS), penicillin-streptomycin (100×) mixture and 0.25% trypsin-EDTA were all purchased from Gibco (USA); the Annexin V-FITC Apoptosis Detection Kit I was purchased from BD Biosciences (USA); the Cell Counting Kit-8 (CCK-8), Cell Cycle and Apoptosis Analysis Kit, DAPI Staining Solution and Triton X-100 were all purchased from Beyotime Biotechnology (China); the Tripoli RNA reagent Kit was purchased from Invitrogen (USA); the Real-time PCR reagent Kit was purchased from KAPA Biosystems (USA).

#### Preparation of NBC

Natural bone ceramic was prepared following the method of Wang et al [30]. In brief, the bovine bone was cut into small cubes, then incubated in 30% H_2_O_2_ solution for 30 min and stewed in a NaOH solution for 1 h to remove collagen and other miscellaneous proteins. After that, the bone cubes were washed with water and immersed in 1% H_3_PO_4_ solution and then heated at 125°C for 2 h. Absolute ethyl alcohol was used to remove moisture. Next, the bone cubes were air-dried for 12 h and heated in a furnace (siliconit muffle furnace, Kwang sung Science Co., Korea) at a rate of 10°C/min up to 1000°C, lasting for 2 h. Finally, the bone cubes were ground and filtered with 120- and 140-screen meshes to obtain NBC particles with a diameter of 105–125 μm.

#### Preparation of composite scaffolds

The type I bovine tendon collagen sponge was dispersed at a concentration of 3% (wt/v) in an acetic acid solution of 0.05 mol/ml, and stirred with a homogenizer (IKA, Germany) in order to get a full dispersion gel. Then, nHA and NBC were, respectively, added into the collagen gel following continuous stirring to obtain the composite hydrogel with a final concentration of 3% (wt/wt). The composite gel was then injected into a 24-well plate and then lyophilized. Collagen gel served as a control. Using this method, three kinds of scaffolds were obtained, CoL, nHA/CoL and NBC/CoL. The scaffolds were fully cross-linked by immersion in a 0.2% (v/v) glutaraldehyde solution (80% ethanol aqueous solution) for 2 h. Finally, the cross-linked scaffolds were freeze-dried and sterilized with 60Co at a dose of 25 kGy.

### Scaffold characterization

#### Scanning electron microscopy

The microstructure and morphology of the nHA and NBC along with their composite scaffolds were characterized by scanning electron microscopy (SEM, JSM 6700 F, Japan) at different magnification with the accelerating potential of 5.0 keV and emission current of 10 mA.

#### X-ray photoelectron spectroscopy analysis

Take a small amount of nHA and NBC powders and sprinkle them on the double-sided tape stuck on the foil paper. Blow the powders into a single layer and cover tape with another tin foil paper, then flatten it with a hydraulic press. Take the samples out, take the double-sided tape down and paste it on the stage. The surface element contents of nHA and NBC were examined using X-ray photoelectron spectroscopy (XPS, ESCALAB 250Xi, USA). Specimens were analysed using a monochromatic Al Kα source (10 mA, 15 kV). The base pressure of the analyser chamber was approximately 5 × 10^−7^ mbar and the electron energy analyser was operated with a pass energy of 20 eV to obtain the high resolution spectra. A step size of 0.05 eV was employed. The elements C, O, P and Ca were established and each peak was scanned twice.

#### Mechanical properties

A Universal Testing Machine (Instron 300DX, USA) was used to carry out measurement of Young’s modulus and compressive tests were executed at a rate of 1 mm/min until the scaffold was crushed. Force and displacement data were recorded in order to generate the stress–strain curves. Young’s modulus was calculated by measuring the slope of the stress–strain curve in the elastic region.

#### Wettability properties

The wettability of the scaffolds was evaluated via contact-angle measurements using an EasyDrop Standard (KRUSS, Hamburg, Germany). Specifically, the freeze-dried scaffolds were whittled into 20 mm × 20 mm × 2 mm cubes with a blade, and then smooth the surface of the cubes with a buffing paper. Finally, press the cubes into a sheet of 0.5 mm thickness with a hydraulic press. Paste the sheets on a glass slide with double-sided tape before test. When testing, a drop of water was dropped from 2 cm above the sheets of material. When the drop contracted on the surface of the scaffold, photographs were captured and the contact angle was calculated.

#### Fourier-transform infrared spectroscopy analysis

The chemical bonds of the composite scaffolds were analysed by Fourier-transform infrared spectroscopy (FTIR, Spectrum GX, USA). For pretreatment, samples were mixed with KBr at a quality ratio of approximately 5% (wt/wt). Transmission IR spectra were set at 4000–400 cm^−1^ with a resolution of 4 cm^−1^, and the scan time was approximately 100 seconds. A pure collagen scaffold served as a reference.

#### X-ray diffraction spectroscopy analysis

The crystal phase composition and distribution of nHA and NBC were analysed by X-ray diffraction spectroscopy (XRD, Smartlab X, Japan). The freeze-dried scaffolds were cut into 20 mm × 20 mm × 2 mm cubes and gently pressed to fill the gas tank. The powder materials directly flatten on the groove of the gas tank as a reference. The XRD test was taken using nickel-filtered Cu *K*α radiation at 40 kV and 200 mA. Spectra were recorded from 2θ = 5°to 90°at a scanning speed of 15°/min and a step size of 0.02°.

### *In vitro* tests

#### Cell seeding and inoculating

The sterilized scaffolds were placed into 24-well (Corning, USA) plates and immersed in α-MEM 3 h before inoculation. MC3T3-E1 cells were seeded in 75 cm^2^ cell culture flasks (Corning, USA) and cultured in α-MEM supplemented with 10% (v/v) FBS and 5% penicillin-streptomycin solution at 37°C in an incubator (MCO-20AIC, Sanyo, Japan) with a 95% air, 5% CO_2_ atmosphere with 100% relative humidity until 80% confluency. Then, the cells were digested using 0.25% trypsin-EDTA. The cell number was calculated using an automated cell counter (Invitrogen^TM^, USA), and the cell suspension was then centrifuged and resuspended in α-MEM (10% FBS) to a final concentration of 1 × 10^6^ cells/ml. The suspension cells were seeded onto the scaffolds placed in 24-well plates with 100 μl added to each scaffold. After 4 h of incubation, 900 μl of medium was added to each well to supplement the final volume to a final 1 ml. The medium was replaced every other day. All cell-based experiments were repeated three times (*n* = 3).

#### Cytotoxicity assay

The cytotoxicity of the scaffolds was evaluated using the CCK8 assay and Annexin V-FITC Apoptosis Detection Kit I [31]. For the CCK8 assay, 100 μl of MC3T3-E1 cell suspension was seeded into the wells of 96-well plates (1 × 10^4^ cells/well). After 4 h, the medium was replaced by material extract that was prepared by soaking the scaffolds in α-MEM (5% FBS) for 24 h in an incubator. Hereafter, 10 μl of CCK8 solution was added into each well and continuously incubated for another 3 h. The culture solution was then transferred to a new 96-well plate, and the optical density (OD) was measured at 450 nm via a microplate reader (infinite M200, Tecan, Swiss). α-MEM (5% FBS) served as a control. For the Annexin V-FITC/PI Apoptosis assay, the cells were collected on days 1 and 3. After seeding, samples were stained with Annexin V-FITC for 30 min at 4°C in the dark, followed by PI staining for 10 min at room temperature in the dark. A flow cytometry system (Accuri^TM^C6, BD Biosciences, USA) was used to detect the survival and death of the cells in the different scaffolds. As validation of the flow cytometry results, a confocal microscope (Olympus IX71, Fluoview, Japan) was also used to observe the day 3 images after staining with Annexin V-FITC/PI.

#### Adhesion and proliferation assays

Cell adhesion morphologies were assessed via SEM at days 1, 3, 7, 14 and 21 after seeding. Briefly, the scaffolds were fixed with 4% (wt/v) Paraformaldehyde at 4°C overnight, dehydrated using an ethanol gradient with 30 min steps each of: 40, 60, 80, 90, 95 and 100% ethanol, critical-point dried, then sputtered with gold and subjected to SEM.

After seeded for 1, 3, 5, 7 and 9 days, the cell proliferation was investigated via CCK8 assay. Briefly, prior to testing, the culture solution of each well was aspirated and 1 ml of fresh α-MEM (10% FBS) was added, followed by the addition of 50 μl of CCK8 solution and incubation for 4 h. Subsequently, 100 μl of the solution was transferred to a new 96-well plate and the OD value was measured at 450 nm via a microplate reader. The CoL group served as a control, and the cell proliferation curve was drawn with culture time as the abscissa and the OD value as the ordinate.

#### MC3T3-E1 differentiation on scaffolds

Each scaffold was divided into two groups, one group was treated with proliferation medium (PM) and the other group was treated with osteogenic-induction medium (OM). In the PM group, MC3T3-E1 cells were cultured in PM containing α-MEM with 100 U/ml penicillin G, 100 mg/ml streptomycin and 10% FBS at 37°C in the incubator [32]. In the OM group, cells were seeded in OM containing 10 mM β-glycerophosphate, 100 nM dexamethasone and 200 μM ascorbic acid to induce osteogenesis [33]. For each group, the medium was replaced every other day. The CoL group served as a control and underwent the same operation as the composite scaffolds groups. Total RNA was extracted and tested on day 14. Briefly, the cells were subjected to total RNA extraction, reverse transcription and real-time quantitative PCR detection, all performed according to the manufacturer’s protocols. The target PCR primers for osteopontin (OPN), bone sialoprotein (BSP), osteocalcin (OCN) and alkaline phosphatase (ALP) were synthesized by Invitrogen (Table 1). β-Actin served as an internal standard. The cycle threshold values (Ct values) were used to calculate the fold differences using the comparative Ct method (also known as the 2^-△△Ct^ method).

**Table 1:** Primer sequences for RT-qPCR

Gene name	Primer ID	Sequence (5′–3′)	Size (bp)
ACTB	Ms-actinF	CGTTGACATCCGTAAAGACC	111
	Ms-actinR	CTAGGAGCCAGAGCAGTAATC	
Osteopontin (OPN)	Q2314	CACTCCAATCGTCCCTAC	127
	Q2315	GTCCTCATCTGTGGCATC	
Bone sialoprotein (BSP)	Q2316	AACGGGTTTCAGCAGACAAC	114
	Q2317	TTCGTTGCCTGTTTGTTCGT	
Osteocalcin (OCN)	Q2318	GACCCTCTCTCTGCTCACTC	123
	Q2319	ACCTTATTGCCCTCCTGCTT	
Alkaline phosphatase (ALP)	Q2322	CGACAGCAAGCCCAAGAG	110
	Q2323	GTGGAGACGCCCATACCA	

### Statistical analysis

Data were analysed using SPSS 17.0 software and represented as the mean±standard deviation. In terms of statistical comparison, the t-test was adopted and after confirmation of homogeneity of variance, one-way analysis of variance as well as the least significant difference (LSD) test was performed. The significance level was taken as α = 0.05, and *P <* *0.05 was considered to indicate statistical significance.

## Results

### XPS analysis

**Figure 1:**
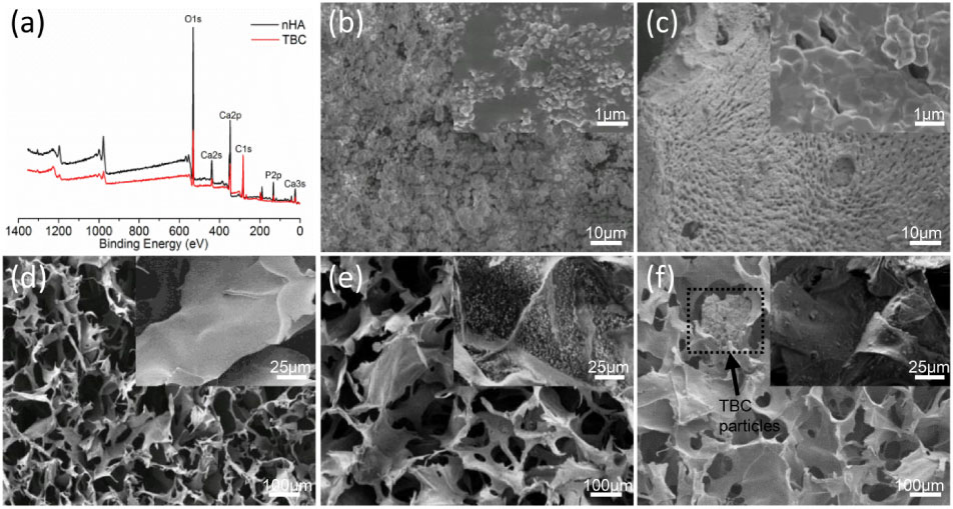
(**a**) X-ray photoelectron spectroscopy spectra of nHA and NBC; (**b**) and (**c**) the SEM images of nHA and NBC (inset to each image is the corresponding pattern, 10 000×). (**d**–**f**) Scanning electron microscopy images of CoL, nHA/CoL and NBC/CoL scaffolds (black dotted box represents the NBC particles; inset to each image is the corresponding pattern, 20 000×)

The results of XPS analysis are shown in Fig. 1a and revealed that the two kinds of calcium phosphate, nHA and NBC, had the same elemental composition, Ca, P, O and C. Calculation of the Ca/P ratio yielded a ratio of 1.67 for nHA and 1.58 for NBC, both of which were consistent with previous reports [20, 34, 35]. That the Ca/P ratio of NBC was lower than that of nHA.

### SEM observation

Scanning electron microscopy was used to observe the surface morphology of the calcium phosphate and scaffold, and the resulting images are shown in Fig. 1. From these images, we observed that there were enormous micro-pores symmetrically distributed on the surface of NBC (Fig. 1c), but this observation was not present in nHA (Fig. 1b). In Fig. 1b and c, the inserted images reveal the nano-form of the two crystals, both of which were spherical in shape and had similar particle diameters. The pore diameters in NBC were approximately 1 μm. The freeze-dried scaffolds presented a 3 D porous structure with pores sizes of approximately 100–300 μm. The CoL scaffold (Fig. 1d) presented an irregular lamellar structure with nonuniform pore distribution, and a tiny deformation of the pores in the collagen scaffold could be seen. The porous structure across the nHA/CoL and NBC/CoL scaffolds (Fig. 1e and f) was regular and exhibited certain rigidity. It was clear that there were NBC particles distributed on the surface of the scaffolds (shown in the black dotted box, Fig. 1f). The images inserted in Fig. 1e revealed that nHA particles were evenly distributed on the surface of collagen in the nHA/CoL scaffold, while the images inserted in Fig. 1f reflected that NBC degradation debris was unevenly distributed on the surface of the NBC/CoL scaffold.

### Mechanical properties (Young’s modulus)

**Figure 2:**
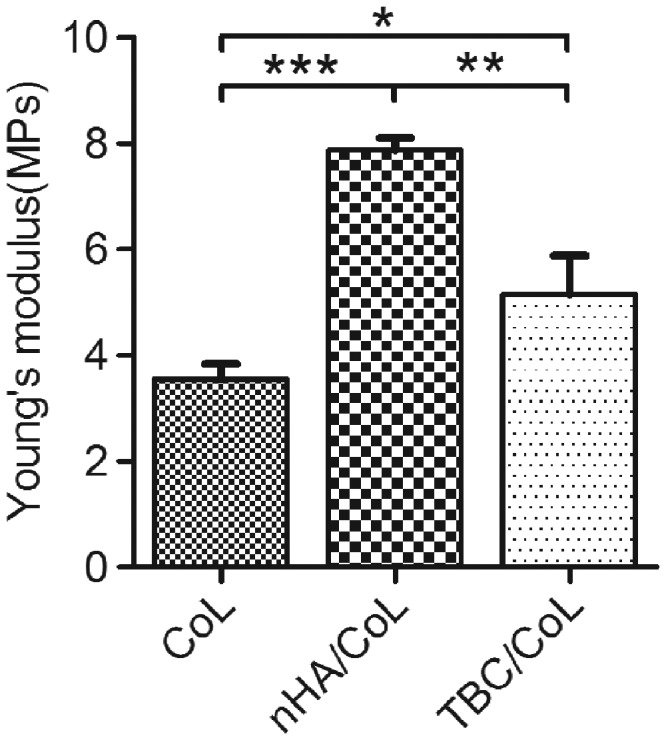
Young’s modulus of the scaffolds (data are mean ± standard, *n* = 3; *P < 0.05, **P < 0.01, **P < 0.01)

Figure 2 shows the Young’s Modulus of the three scaffolds. The result displayed that the Young’s Modulus measurement of the nHA/CoL group was 7.9 ± 0.2 MPa, which was higher than those of the CoL and NBC/CoL groups, which were 3.5 ± 0.3 MPa (**P <* *0.01) and 5.1 ± 0.7 MPa (*P <* *0.05), respectively.

### Wettability properties

**Figure 3:**
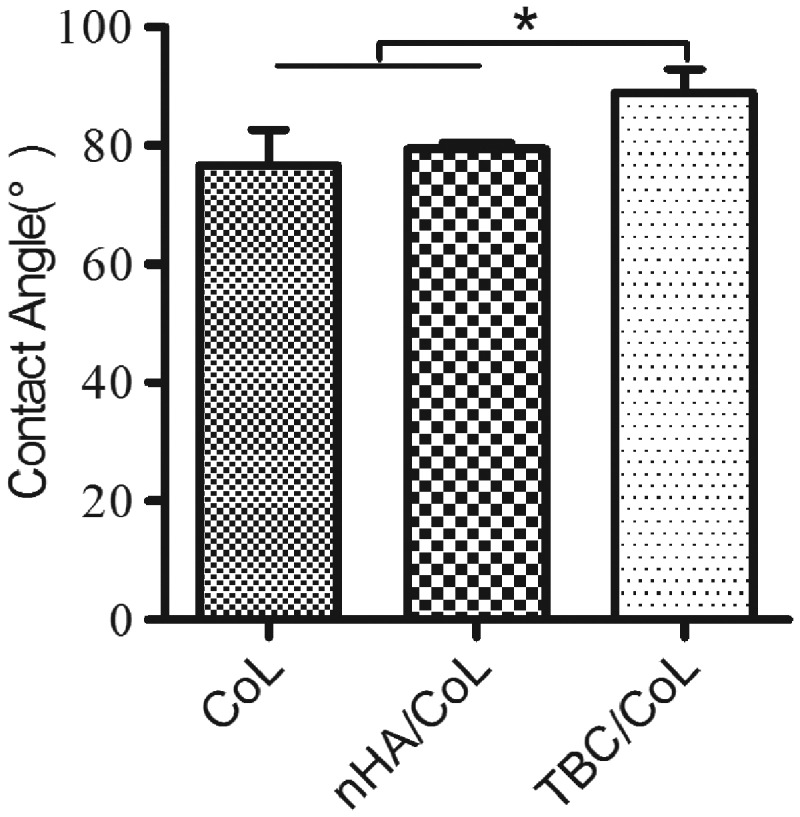
Water contact-angle analysis (data are mean ± standard, *n* = 3; *P < 0.05)

The water contact angle (wettability) of the scaffolds, are shown in Fig. 3, indicated that there were no significant differences between the two scaffolds, CoL and nHA/CoL, for which the average water contact angles were 76.7 ± 5.8° and 79.4 ± 0.9°, respectively. However, the contact angle for NBC/CoL was 88.7 ± 4.1°, higher than that of the other two groups (*P <* *0.05).

### FTIR analysis

**Figure 4:**
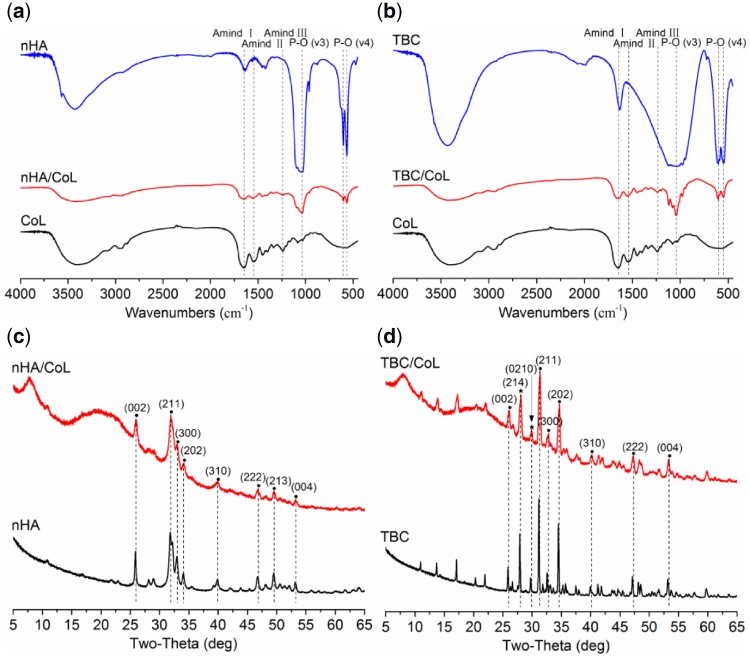
Fourier-transform infrared spectroscopy patterns of nHA and nHA/CoL (**a**), and NBC and NBC/CoL (**b**); dashed lines indicate the similar symbolic peaks of the two groups: 1037 cm^−1^: *ν_3_*vibration of ${\text{PO}}_{4}^{3-}$; 606 cm^−1^ and 562 cm^−1^:*ν_4_*vibration of ${\text{PO}}_{4}^{3-}$; 1655 cm^−1^: amideI; 1550 cm^−1^: amide II; 1237 cm^−1^: amide III. X-ray diffraction patterns of nHA and nHA/CoL (**c**) and NBC and NBC/CoL (d); the black dots represent for the Ca_10_(PO_4_)_6_(OH)_2_ phases; The black pentagrams in (**d**) represent the β-TCP phases; the dashed lines indicate the similar crystal phase diffraction peaks of two materials

The FTIR spectra of the scaffolds are shown in Fig. 4a and b. The peaks at 3568.2 cm^−1^ and 3440.5 cm^−1^ belonged to the stretching vibration of –OH [36]. The peaks at 1037, 603 and 567 cm^−1^ were the major bonds associated with ${\text{PO}}_{4}^{3}$^−^ and appeared in both nHA and NBC [37]. The peak at 1037 cm^−1^ represented the stretching modes of the P-O (ν_3_) bonds, while the doublet at 606 cm^−1^ and 562 cm^−1^ was due to the bending modes of the P-O (ν_4_) bonds in HA [38]. The main bands of collagen arose from peptide bond vibrations: amides A, I, II and III. The peaks at 3330 and 3428.6 cm^−1^ belonged to the N = H stretching of amide A. The peak at 1655 cm^−1^ was the C = O absorption of the amide, 1550 cm^−1^ was the C-N stretching and C-H bending combination vibration, both of which belonged to the amide. The peak at 1237 cm^−1^ was a sign of C-N stretching, N-H bending and C-C stretching, which belonged to the amide [39]. From the FTIR spectra, the two groups of scaffolds were consistent in the vibration of ${\text{PO}}_{4}^{3}$^−^ at 1037, 603 and 567 cm^−1^. Nevertheless, the peaks at 970 and 1127 cm^−1^ in the NBC/CoL scaffold were ${\text{PO}}_{4}^{3}$^−^ absorption of β-TCP [37], which among the spectra, only presented in those of NBC and NBC/CoL.

### XRD analysis

The XRD spectra of the nHA and NBC along with their composite scaffolds are shown in Fig. 4c and d. To analyse the crystalline phase compositions, we checked the HA phases incorporated in the International Centre for Diffraction Data (ICDD) databases that showed the XRD patterns of HA. The results indicated that the crystalline phases in nHA and NBC were for the most part consistent with the Ca_10_(PO_4_)_6_(OH)_2_ phase (ICDD No.41-0487) [10, 40]. The peaks at 8° and 21.08° in the spectra were the diffraction peaks of the amorphous structure of collagen [41]. The main miller indices for TCP are in (024), (214) and (0210), which mainly presented in NBC. Miller indices in (002), (211), (202), etc. represented the characteristic diffraction peak of HA [20]. These XRD results were in accordance with the finding of investigation of FTIR that TCP phases only exist in crystalline NBC. Moreover, the positions of the main diffraction peak of nHA and NBC were not changed after combination with collagen, but the peak widths were increased.

### Cytotoxicity evaluation

**Figure 5:**
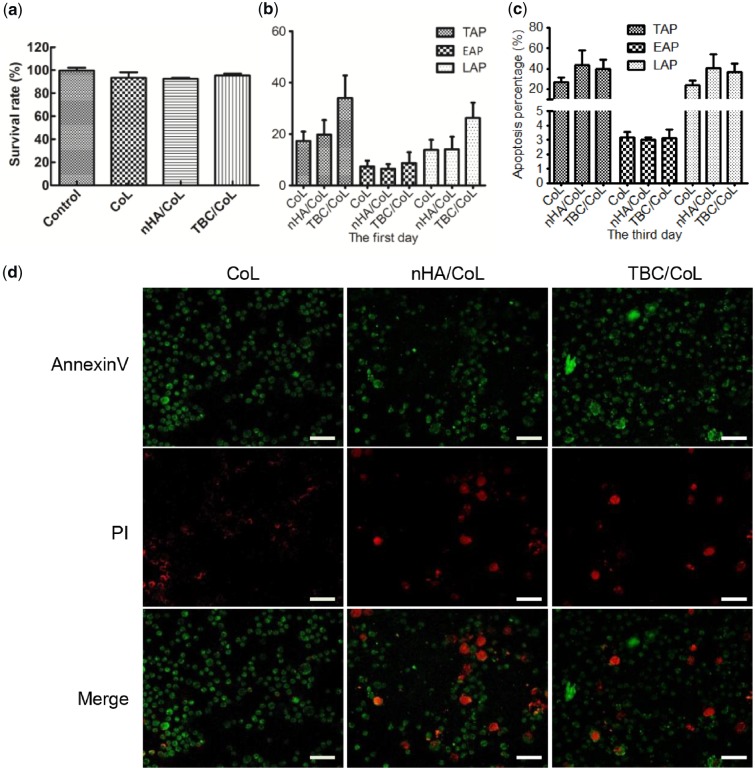
(**a**) CCK8 assay for cytotoxicity of control and conditioned media prepared from scaffolds (1 day incubation) or control media toward MC3T3-E1cells. (**b**) and (**c**) Assessment of MC3T3-E1 survival and apoptosis for and 3 days after seeding on the scaffolds using annexin V-FITC/PI double-staining assay. (TAP represents total apoptotic percentage; EAP represents early apoptotic percentage; LAP represents late apoptotic percentage.) (**d**) Apoptosis staining after 3 days of culture on the scaffold. (dark red represents late apoptotic cells and light red represents early apoptotic cells) (40×, scale bar = 100 μm)

The results of cytotoxicity testing are shown in Fig. 5. The CCK8 test (Fig. 5a) showed that the average cell survival rate of the three groups was greater than 90%, indicating that the scaffold extract has no cytotoxicity against MC3T3-E1 when compared to the control medium [42]. Additionally, flow cytometry analysis revealed that there was no significant difference in the number of survived and dead cells between the composite scaffolds and the control group (Fig. 5b and c). The results of Annexin V-FITC/PI staining (Fig. 5d) of MC3T3-E1 directly reflected the apoptosis of cells cultured in the scaffold at day 3, which was consistent with the findings of the flow cytometry studies.

### MC3T3-E1 adhesion on scaffolds

**Figure 6:**
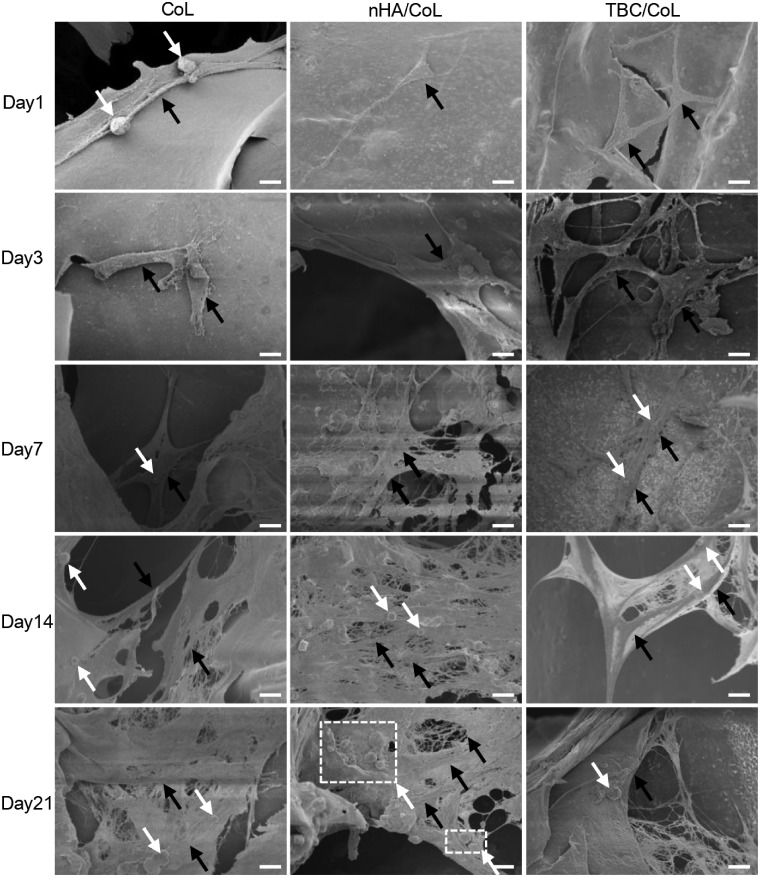
Scanning electron microscopy images of MC3T3-E1 cells on the surface of the scaffolds on days 1, 3, 7, 14 and 21 after seeding (black arrows indicate the cells; white arrows and white square frames indicate the extracellular secretions of MC3T3-E1; scale bar = 100 μm)

We used SEM in order to observe the cell adhesion and proliferation on the scaffolds (Fig. 6). The SEM images showed that MC3T3-E1 cells were firmly adhered to the scaffolds after 24 h of seeding, and had formed numerous cellular pseudopods attaching to the surface of scaffold. On day 3, we found that the cells were compatible with the scaffolds, and had already divided or were currently dividing into daughter cells. On day 7, we observed that the cells could step across the scaffold and showed the trend of the three-dimensional growth style. On days 14 and 21, the scaffolds were partially or fully covered by cells. Furthermore, on day 14, the cells had begun to grow in stacks in the nHA/CoL scaffold. On day 21, numerous extracellular secretions could be seen on the surface of the cell clusters, especially in the nHA/CoL scaffold, as marked by white square frame in nHA/CoL group. Moreover, due to degradation, the scaffold partially presented a mesh structure.

### MC3T3-E1 proliferation on scaffolds

**Figure 7:**
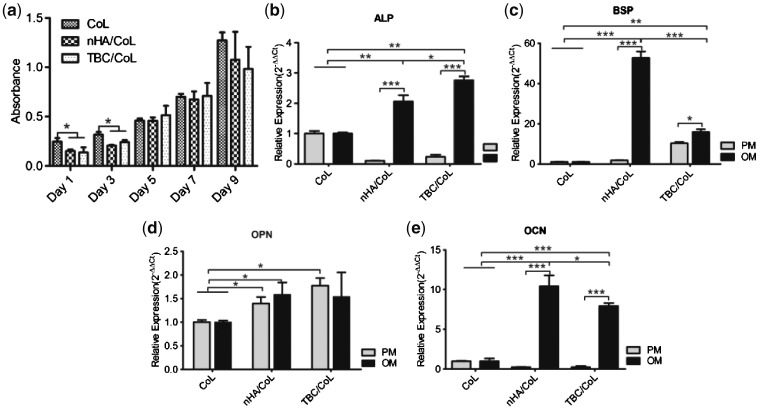
(**a**) Measurement of MC3T3-E1 proliferation on the scaffolds using the CCK8 assay at 1, 3, 5, 7 and 9 days after seeding; (**b**–**e**) expression of ALP, BSP and OCN genes in MC3T3-E1 cells cultured in PM or OM. (*P < 0.05, **P < 0.01, ***P < 0.01, *n* = 3)

The CCK8 test was used to evaluate the proliferation of cells on the scaffolds, on days 1, 3, 5, 7 and 9 (Fig. 7a). The OD values for all groups increased with time. On days 1 and 3, the OD values observed for the CoL scaffold group were significantly higher than those in the composite scaffold groups (*P <* *0.05), while on days 5, 7 and 9, there was no significant difference between the three groups.

### MC3T3-E1 differentiation on scaffolds

The MC3T3-El cells seeded on the scaffold were cultured in OM or PM medium for 14 days, and then the mRNA transcription levels of the osteo-related genes of ALP, BSP, OPN and OCN were examined by RT-PCR (Fig. 7b–e). The results revealed that, in the composite scaffold group in OM, there was a significant increase in the expression levels of the genes ALP, BSP and OCN when compared with the PM group, as well the CoL group cultured both in OM and PM.

Expression of the genes BSP and OCN was higher in the nHA/CoL group in OM than in the NBC/CoL group in OM, whereas the expression of ALP and OPN was higher in the NBC/CoL group than in the nHA/CoL OM group. Further, expression of the OPN gene in the composite scaffold was higher than that in the CoL scaffold.

## Discussion

Hydroxyapatite is one of the main inorganic components of bone and consists primarily of calcium phosphates, the primary raw material for synthetic bone [40]. Two forms of HA, nHA and NBC, offer promising biological advantages as bone analogs and have been widely applied clinically as bone graft substitutes [22, 43–46]. In this study, we prepared two kinds of composite scaffolds using nHA and NBC mixed with collagen. There are several potential roles for nHA or NBC in the composite scaffold, such as serving as a water-wetting media, a reinforcing phrase and the stimulating factor for osteogenesis [47]. In order to enhance mechanical properties, slow down enzyme degradation and maintain the structure of the scaffolds, glutaraldehyde was used as a cross-linker [48].

In our hypothesis, we posed that the morphological and structural differences between nHA and NBC might cause a diversity of chemo-physical properties that could lead to different proliferation and differentiation properties of cells *in vitro* as well as for later clinical application. In this research, we detected several chemo-physical and biological properties in order to evaluate the scaffolds.

The results of our XPS analysis showed that NBC had a lower Ca/P ratio than nHA, which indicate that the Ca_10_(PO_4_)_6_(OH)_2_ in calf bone partially might converted to β-Ca_3_(PO_4_)_2_ during the calcination process [20]. The XPS analysis further verified that TCP phases only existed in NBC, illustrating that during the process of calcination the bovine bone might have decomposed into TCP and HA [30]. It has been reported that apatite in natural bone is a type of HA with an imperfect crystal structure, closer to that of calcium-deficient HA which has a Ca/P of about 1.5, similar to Ca/P in TCP [49, 50]. Moreover, it has been reported in the literature that, *in vivo*, NBC degrades and resolves faster than HA due to the high biodegradation rate and solubility of β-TCP [51].

The SEM reflected the surface morphology of the calcium phosphate and scaffold. From the images, nHA evenly distributed on the surface of nHA/CoL scaffold, while NBC degradation debris could be seen unevenly distributed on the surface of NBC/CoL. (The images inserted in Fig. 1e and f.) The NBC debris might be caused by the acid dissolution and mechanical stirring. HA particle size and distribution density on the scaffold might cause the differences in the hydrophilicity of the scaffolds and affect the mineralization of collagen [31, 52]. In natural bone, collagen fibers provide a matrix for the bone, around which HA crystals form in an oriented arrangement. Ca^2+^ released from HA coordinates with fibers from sugar hydroxyl, which causes collagen mineralization and greatly increases mechanical properties [53]. Thus, the mineralization of collagen in the composite scaffolds was an important detection factor. In this study, we used FTIR and XRD to analyse the degree of collagen mineralization in the two composite scaffolds. From the FTIR spectra, the existence of amides A, I, II and III indicated that the collagen triple helix structure was well maintained during the scaffold preparation [54, 55]. The amide A vibrations weakened and redshifted, which might have been caused by the formation of coordination bonds of amino and Ca^2+^ iron though electrostatic or chemical adsorption complexation. Vibration of amides I, II and III in the composite scaffold weakens substantially, indicating that apatite has banded into the collagen nucleation site, causing the weakening of absorption peaks [53]. In particular, the amide absorption band in nHA/CoL had a weaker absorption than in NBC/CoL. Hence, we concluded that the reason why the mechanical strength of NBC/CoL was lower than that of nHA/CoL might be that the low rate of Ca^2+^ released by the lower specific surface area of NBC resulted in poor binding ability to collagen, which presented nHA/CoL had a more outstanding mechanical property, as shown in Fig. 2. From the XRD spectra, we observed that the HA crystal diffraction peak of the two composite scaffolds had broadened compared to that of calcium phosphate in the same position, which might be due the orientation effect of the crystal phase induced by collagen [30, 53]. In a word, CoL has the effect of inducing the orientation of disaggregated crystalline HA from nHA or NBC during the preparation of the scaffold [56].

The CCK8 assay demonstrated that the scaffolds were non-toxic to MC3T3-E1 cells *in vitro*. Furthermore, apoptosis detection and Annexin V-FITC/PI staining reflected that the apoptosis rate of cells cultured in the NBC/CoL and nHA/CoL scaffolds were not significantly different from those cultured in CoL scaffolds, indicating that nHA and NBC posed no obvious cytotoxicity to MC3T3-E1 cells *in vitro*. For the relatively high apoptosis rates (about 40%) in the experimental groups, we believed that there are two reasons. First, since it was hard to completely dissociate the cells from the scaffolds by enzymatic hydrolysis with 0.25% trypsin, we performed a second enzymatic treatment. However, the dissociation time and frequency might lead to a high apoptosis rate. Second, Ca^2+^ released from HA might affect the physiological properties of cells and thus affect its apoptosis. Ca^2+^ act as a second messenger in body to participate in various cellular metabolism and hormone metabolism, and can also regulate the permeability of cell membranes. In our study, Ca^2+^ released from the scaffolds might increase the permeability of the cell membrane, which led to the entry of PI molecules into the cell and bonded to the nucleus, thus resulted to a higher late apoptosis rate of cells.

Scanning electron microscopy images revealed the specific morphology and adhesion of MC3T3-E1 cells at different days on the scaffold. The cell density reflected the increasing proliferation of MC3T3-E1 cells on the scaffold at different days, which was consistent with the results of the CCK8 assays. From the images, it could be seen that the cells adhered to the wall as well as the ridges of the scaffold, and had grown inside until they completely enveloped it. Extracellular secretions could also be seen clearly dispersed around the cells. Following 14 days of culture, the scaffold displayed a degradation performance. These aforementioned properties reflected the good *in vitro* cell compatibility and degradability of the scaffold.

The findings from both the CCK8 assay and SEM indicated that the scaffolds have good cytocompatibility to MC3T3-E1 cells. We found that cells in CoL had a quicker metabolism and higher OD value at an early period of time after seeding, which could be due to the better hydrophilic property of the CoL scaffold, lending a better affinity to MC3T3-E1 cells. As seen in Fig. 7a, after 5 days, the OD values of the three groups were at the same level, while on day 9, the absorbance of the composite scaffold groups had decreased, possibly due differentiation rather than proliferation of the cells in the two scaffolds [57].

When examining the osteo-promoting effects of nHA and NBC on MC3T3-E1 cells on the 3 D scaffold, we found that both nHA and NBC promoted osteogenic differentiation, a finding that was consistent with previous studies [30, 51]. Osteopontin is an extracellular structural protein and an organic component of bone [52]. Its gene was expressed stably in the early stage process, and with the help of other extracellular matrix proteins, this protein could connect all cells together [58]. Alkaline phosphatase is a bone marker protein that matures in the early extracellular matrix and is one of the enzymes necessary for ossification in the cell. Alkaline phosphatase could accelerate cellular phosphorylation and promote significant mineralization of extracellular matrix. Furthermore, bone sialoprotein and osteocalcin are marker proteins for late osteogenic differentiation. BSP is a gene associated with mineralization that is a marker for specific and late osteoblast differentiation [59]. Meanwhile, OCN is secreted solely by osteoblasts and is also implicated in bone mineralization and calcium ion homeostasis [60].

Our results showed that expression of the osteo-related genes ALP, BSP and OCN in the nHA/CoL and NBC/CoL OM groups was significantly higher than in the PM group as well as all of the CoL groups. The expression of OPN in both the nHA/CoL and NBC/CoL PM groups was higher than in the CoL PM group. It seemed that HA was able to promote OPN gene expression, and that this promoting effect was not affected by osteogenic differentiation factor. The RT-PCR investigation illustrated that both nHA and NBC could promote MC3T3-E1 osteoblast mineralization, while NBC tended to promote early osteogenic gene expression, and nHA preferred to upregulate late osteogenic gene expression. Nevertheless, more research needs to be done to prove this standpoint. So far, little is known about the mechanism by which HA promotes osteogenesis. It has been reported that HA has the capacity to lead sustained changes in the expression of the osteoblast differentiation marker genes through DNA methylation, allowing for the passage of changes to daughter cells during division [61]. This could be a reason for the significant upregulation of osteogenic genes in the nHA/CoL and NBC/CoL OM groups over those in CoL group in this study. Moreover, Ca^2+^ was more easily released from the nHA particles due to its smaller surface area, which could explain the higher expression of osteogenic genes in the nHA/CoL scaffold (than in NBC/CoL scaffold), but this finding still requires more experimental validation. In a word, HA could alter osteoblast behavior through specific molecular actions, and then go on to promote the osteogenesis of MC3T3-E1 cells. However, more research is necessary in order to fully explore the detailed mechanism of HA’s osteo-promoting character as well as that of nHA and NBC osteo-promoting character and the rules by which they interact.

## Conclusions

In this study, we prepared two composite collagen scaffolds, nHA/CoL and NBC/CoL, by homogeneously dispersing nHA and NBC in type I collagen. The detection results showed that both of the two composite scaffolds had the potential to promote osteogenic differentiation of MC3T3-E1 cells. The NBC ceramic, consisting of HA and TCP, could promote early osteogenic gene expression, while nHA had the potential to upregulate the late osteogenic gene. Future studies are required in order to explore the specific mechanism of osteo-promoting differentiation between nHA and NBC.
